# Editorial Note: *Sclerotium rolfsii* Lectin Induces Stronger Inhibition of Proliferation in Human Breast Cancer Cells than Normal Human Mammary Epithelial Cells by Induction of Cell Apoptosis

**DOI:** 10.1371/journal.pone.0361930

**Published:** 2026-11-03

**Authors:** The PLOS One Editors

The *PLOS One* Editors issue this Editorial Note to inform readers that concerns have been raised about results presented in Fig 7B of this article [1].
